# Nomogram for predicting risk factor of urosepsis in patients with diabetes after percutaneous nephrolithotomy

**DOI:** 10.1186/s12871-022-01629-1

**Published:** 2022-03-31

**Authors:** Jie Gu, Jun Liu, Yang Hong, Yi Feng, Xiaobo Huang

**Affiliations:** 1 Department of Anesthesiology, Peking University People’s Hospital, 133 Fuchengmen Inner Street, Xicheng District, Beijing, 100034 People’s Republic of China; 2 Urology and Lithotripsy Center, Peking University People’s Hospital, 133 Fuchengmen Inner Street, Xicheng District, Beijing, 100034 People’s Republic of China; 3 Peking University Applied Lithotripsy Institute, Peking University, 100034 Beijing, People’s Republic of China

**Keywords:** Percutaneous nephrolithotomy (PCNL), Upper urinary calculi, Urosepsis, Diabetes, Nomogram

## Abstract

**Background:**

Urosepsis is an infectious complication after percutaneous nephrolithotomy (PCNL). This study aimed to analyze the perioperative factors related to urosepsis after PCNL for upper urinary calculi and establish a nomogram to predict the probability of postoperative urosepsis based on the risk factors.

**Methods:**

The Clinical data of one-stage PCNL for upper urinary stones in patients already diagnosed with type 2 diabetes between June 2010 and June 2020 were retrospectively analyzed. The patients were divided into two groups according to whether urosepsis occurred after surgery, and univariate and multivariate logistic regression analyses evaluated the risk factors for urosepsis. Moreover, the corresponding nomogram prediction model was determined by the regression coefficient.

**Results:**

All 366 patients with diabetes underwent one-stage PCNL. Seventy-one (19.4%) patients had urosepsis after surgery, and their hospitalization time was longer than that of patients without urosepsis. Moreover, the incidence of non-infection-related complications was higher. Multivariate logistic regression analysis revealed four independent risk factors associated with postoperative urosepsis, including positive urine nitrite (odds ratio [OR] = 3.326, *P* = 0.007), positive urine culture (OR = 2.213, *P* = 0.023), intraoperative hypotension (OR = 8.968, *P* < 0.001), and staghorn calculi (OR = 3.180, *P* = 0.002). The above independent risk factors were used as variables to construct the nomogram. The nomogram model was internally validated. The calculated concordance index was 0.824. The Hosmer–Lemeshow goodness-of-fit test was performed (*P* = 0.972 > 0.05). The area under the curve of this model was 0.831, indicating that the nomogram model had good accuracy in predicting the probability of urosepsis in patients who underwent PCNL with diabetes and had good consistency with the actual risk.

**Conclusion:**

Positive urine culture, positive urine nitrite, staghorn calculi, and intraoperative hypotension were independent risk factors for urosepsis in patients who underwent one-stage PCNL with diabetes. The new nomogram could accurately assess the risk of urosepsis after PCNL in patients with diabetes.

## Background

Percutaneous nephrolithotomy (PCNL) has become the first choice for patients with upper urinary tract stones, especially kidney stones or upper ureteral stones with a diameter greater than 2 cm [1]. Notably, urosepsis is one of the most serious complications after PCNL and may be life-threatening if not treated promptly or treated improperly [2]. Studies have shown that PCNL in patients with diabetes has a 14.6-fold higher risk of postoperative infectious complications than that in patients without diabetes [3]. Thus, early identification of patients at potential risk of urosepsis when performing PCNL for patients with diabetes is considered clinically significant.

Many studies have demonstrated the risk factors for urosepsis after PCNL [4, 5], including staghorn calculi, positive midstream urine culture, preoperative stenting, and bladder outlet obstruction. However, at present, most studies ignore an important factor, namely intraoperative blood pressure, combined with intraoperative anesthetic blood pressure. Thus, this study aimed to provide more effective and reliable clinical parameters and construct a prediction model of postoperative urosepsis to help urologists in the early assessment of the probability of perioperative urosepsis in patients with diabetes undergoing PCNL, followed by early prevention and intervention.

## Methods

### Study population

The Clinical data of one-stage PCNL for upper urinary stones in patients already diagnosed with type 2 diabetes at the Urology and Lithotripsy Center of Peking University People’s Hospital between June 2010 and June 2020 were retrospectively analyzed. The exclusion criteria were as follows: [1] history of bilateral endoscopic lithotripsy for one-stage PCNL; [2] life-threatening or even more severe systemic diseases, that is, American Society of Anesthesiologists (ASA) status IV–VI; [3] solitary kidney and history of nephrectomy; and [4] missing data. The study conformed to the principles of the 1964 Declaration of Helsinki and was conducted in accordance with the ethical standards of the medical ethics committee of our institution.

### Data collection

We recorded the information in an electronic anesthesia record system certified by the Association for Healthcare Information and Management Systems, and our data were all obtained from an electronic database on the server of our institution. We collected demographic data, including age, sex, body mass index (BMI), and ASA status. Clinical data included relevant preoperative examinations and surgery-related information.

Preoperative examination included blood routine, serum biochemistry, and kidney ultrasound and abdominal radiography, or urinary computed tomography was performed. Midstream urine from all patients underwent urinalysis and urine bacterial culture. A positive urine white blood cell (WBC) count was defined as a WBC count ≥ 10 per high-power field (× 400). For patients with positive urine WBC or positive urine nitrite, antibiotics were immediately administered for 3–7 days and adjusted according to urine culture results and drug sensitivity testing during treatment. All patients were given antibiotic prophylaxis 30 min before surgery and continued 48 h after surgery if urine culture was positive. The operation time, intraoperative blood pressure and heart rate, postoperative respiratory rate, heart rate, body temperature, blood pressure, blood WBC count, and serum creatinine level were recorded.

All patients were followed up postoperatively. We used the systemic inflammatory response syndrome (SIRS) criteria (WBC count 4000 × 10^9^/L or > 12,000 × 10^9^/L; fever > 38 °C or < 36 °C; heart rate > 90 beats/min; respiratory rate > 20 breaths/min), and blood cultures were performed if necessary [6]. The diagnosis of SIRS was established when a patient met two or more of the above criteria. The diagnostic criteria for urosepsis are simultaneous urinary tract infection and SIRS and exclusion of infections at other sites [7].

The definition of intraoperative hypotension remains controversial. Based on several previous studies [2, 8], we used these thresholds in our study: after induction of anesthesia, systolic blood pressure was ≤ 90 mmHg or it decreased by ≥ 30% from pre-induction (mean arterial pressure), and lasted more than 5 min.

Intraoperative and 30-day postoperative surgical complications were recorded and classified according to the modified Clavien system [9].

Diagnosis of type 2 diabetes mellitus (T2DM) was defined as a 2-h plasma glucose (PG) (2-h 75-g postload PG) level ≥ 11.1 mmol/L on oral glucose tolerance test or fasting PG level ≥ 7.0 mmol/L according to the American Diabetes Association criteria, or a previous diagnosis of T2DM.

### Percutaneous nephrolithotomy

All patients received spinal anesthesia (spinal or combined spinal anesthesia) in the lithotomy position. Moreover, a 5-Fr ureteral catheter (Bard, USA) was placed on the affected side under cystoscopy, the urinary catheter was placed, patients were placed in the prone position, and percutaneous access was obtained under ultrasound guidance. Subsequently, with the guidance of the guide wire, a 24-Fr Amplatz sheath was inserted for nephrostomy access. Moreover, a 20.8-Fr rigid nephroscope (Richard Wolf, Knittlingen, Germany) combined with ultrasound and pneumatic lithotripsy (Swiss lithotriptor, EMS, Nyon, Switzerland) was used for stone removal in all patients. At the end of the operation, a 6-F double J stent and 20-F nephrostomy tube were placed for drainage, which were usually retained in the body for 2–4 weeks and 3 days, respectively.

### Statistical methods

The data were analyzed using the Statistical Package for the Social Sciences software (version 20.0). Data are presented as mean ± standard deviation or median and range. Student’s t-test was used to compare continuous variables with normal distribution, and the groups were compared using the Mann–Whitney U test. The chi-square test was used to analyze the comparison between groups of disordered classified variables, and the risk factors were analyzed by univariate and multivariate analyses and logistic regression analysis. Statistical significance was set at *P* < 0.05.

According to the results of the logistic regression analysis model, the nomogram prediction model was constructed using the R software (R3.6.2 Institute of Statistics and Mathematics, Vienna, Austria) rms package. The bootstrap method was used for repeated sampling 1000 times to internally validate the nomogram model, and a discrimination test was performed by calculating the consistency index (C-index) and area under the curve (AUC), drawing the calibration curve, and performing the Hosmer–Lemeshow goodness-of-fit test to assess the model accuracy.

## Results

### Clinical data and surgical outcome

**Fig. 1:**
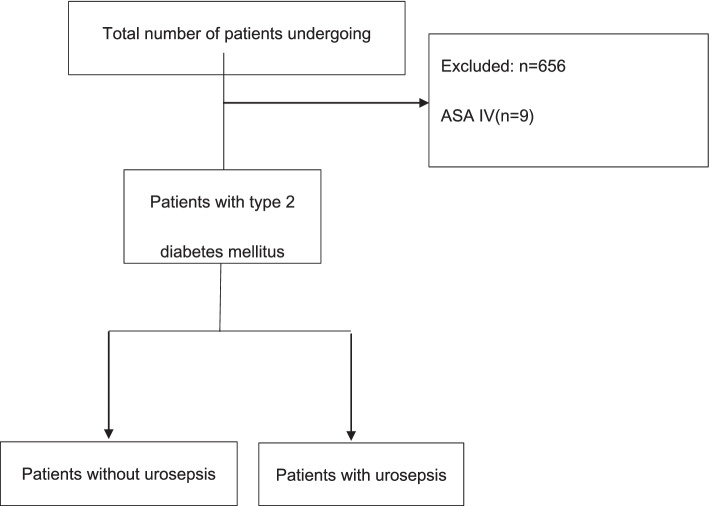
Patient flowchart

A total of 366 patients with detailed clinical data were consecutively enrolled and divided into the urosepsis and non-urosepsis groups according to the occurrence of urosepsis (Fig. 1).

One-stage PCNL was successfully performed in 366 (218 male and 148 female) patients with diabetes (age 28–81 [mean age, 57.8] years; mean stone size, 2.85 ± 2.11 cm; and operation time, 70 [20–175] min). After the operation, 71 (19.4%) patients had urosepsis, which occurred within 24 h after the operation and improved after active antibiotic treatment. Three patients were admitted in intensive care unit, and no deaths were observed. According to whether urosepsis occurred after operation, the patients were divided into the urosepsis and non-urosepsis groups. Table 1 shown the details of clinical data in the two groups. Compared with the non-urosepsis group, the urosepsis group had more patients with positive urinary nitrite preoperatively (*P* < 0.001) and longer hospital stay (12.6 ± 4.9 vs. 10.5 ± 4.5, *P* < 0.001). Female patients had a higher incidence of postoperative urosepsis than male patients (*P* < 0.002), among patients with staghorn calculus, residual stones during operation, and hypotension during operation, the incidence of urosepsis after surgery was higher in the urosepsis group than in the control group (*P* < 0.05). Notably, there were no significant differences in age, BMI, blood glucose level, operation time, number of channels between the two groups.

**Table 1:** Comparison of clinical data between patients with and without postoperative urosepsis

Items	Non-urosepsis	Urosepsis	*P* value
Sex, n (%)			
Male/ Female	187/108	31/40	0.002
Age/years	57.4 ± 9.9	59.7 ± 9.3	0.077
BMI (kg/m^2^)	26.3 ± 3.8	25.7 ± 4.2	0.235
Hypertension			
Yes/No	178/117	41/30	0.690
Staghorn stone			
Yes/No	39/256	26/45	< 0.001
Preoperative urine WBC count			
Positive/Negative	20/275	7/64	0.374
Preoperative urine nitrite level			
Positive/Negative	24/271	20/51	< 0.001
Preoperative urine culture			
Positive/Negative	101/194	41/30	< 0.001
Preoperative hemoglobin level (g/L)	134.6 ± 19.6	130.4 ± 18.9	0.101
Preoperative serum creatine			
level (μmol/L)	89.6 ± 54.3	94.7 ± 74.3	0.586
Preoperative blood glucose level			
(mmol/L)	7.0 ± 2.1	7.1 ± 2.1	0.532
ASA status			
I–II/III	279/16	64/7	0.167
Operative time/min	75.2 ± 39.1	71.8 ± 26.3	0.378
Tract			
Single/Multiple	270/25	64/7	0.711
Stone clearance			
Yes/No	32/263	57/14	0.043
Intraoperative hypotension			
Yes/No	43/252	43/28	< 0.001
Hospital stay (days)	10.5 ± 4.5	12.6 ± 4.9	< 0.001
Complication			
0	263	54	
I–II/III–IV	20/12	14/3	
Total	32	17	0.004

*BMI* Body Mass Index, *WBC* White Blood Cell, *ASA* American Society of Anesthesiologists

### Logistic regression analysis and risk factors

In the univariate analysis, we analyzed the potential predictors from the following three aspects: patient condition, stone, and surgery (Table 2). The results showed that the factors significantly related to postoperative urosepsis in univariate analysis included sex (odds ratio [OR] = 2.234, *P* = 0.003), preoperative urinary culture results (OR = 2.612, *P* < 0.001), urinary nitrite (OR = 4.395, *P* < 0.001), staghorn stone (OR = 3.793, *P* < 0.001), and intraoperative hypotension (OR = 9.000, *P* < 0.001). The results showed that as independent risk factors for postoperative urosepsis, including preoperative urinary nitrite positivity (OR = 3.326, *P* = 0.007), urinary culture positivity (OR = 2.123, *P* = 0.023), staghorn calculi (OR = 3.180, *P* < 0.002), and intraoperative hypotension (OR = 8.968, *P* < 0.001).

**Table 2:** Univariate and multivariate logistic regression analyses of clinical data and urosepsis

Variables	Univariate analysis			Multivariate analysis		
	OR	95% CI	*P* value	OR	95% CI	*P* value
Sex					0.584–2.304	
Female/male	2.234	1.321–3.778	0.003	1.160		0.672
Age (> 65)	1.402	0.772–2.544	0.274			
BMI (> 28)	0.719	0.390–1.324	0.280			
Preoperative blood glucose (mmol/L, > 6.1)	1.142	0.665–1.961	0.628			
Preoperative serum creatine (μmol/L, > 84)	0.901	0.524–1.547	0.705			
Preoperative urine nitrite	4.395	2.261–8.544	< 0.001	3.326	1.397– 7.920	0.007
Preoperative urine culture	2.612	1.539–4.432	< 0.001	2.123	1.110–4.059	0.023
Preoperative blood WBC (≥ 10 × 109/L)	1.504	0.610–3.709	0.376			
OR time (≥ 90 min)	1.092	0.630–1.891	0.754			
ASA grade	1.907	0.753–4.828	0.190			
Intraoperative hypotension	9.000	5.062–16.001	< 0.001	8.968	4.724–17.025	< 0.001
Staghorn stone	3.793	2.105–6.833	< 0.001	3.180	1.515–6.677	0.002
Stone clearance	0.532	0.244–1.158	0.124			

### Construction of nomogram for prediction of urosepsis

**Fig. 2:**
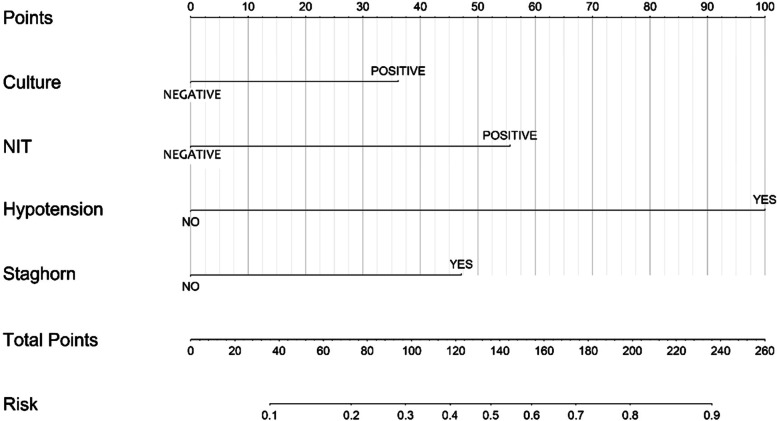
Nomogram for predicting the probability of postoperative urosepsis following PCNL in patients with DM. Culture: Preoperative urine culture; NIT: Preoperative urine nitrite; Hypotension: Intraoperative hypotension; Clearance: Stone clearance. DM = diabetes mellitus. PCNL = percutaneous nephrolithotomy

**Fig. 3:**
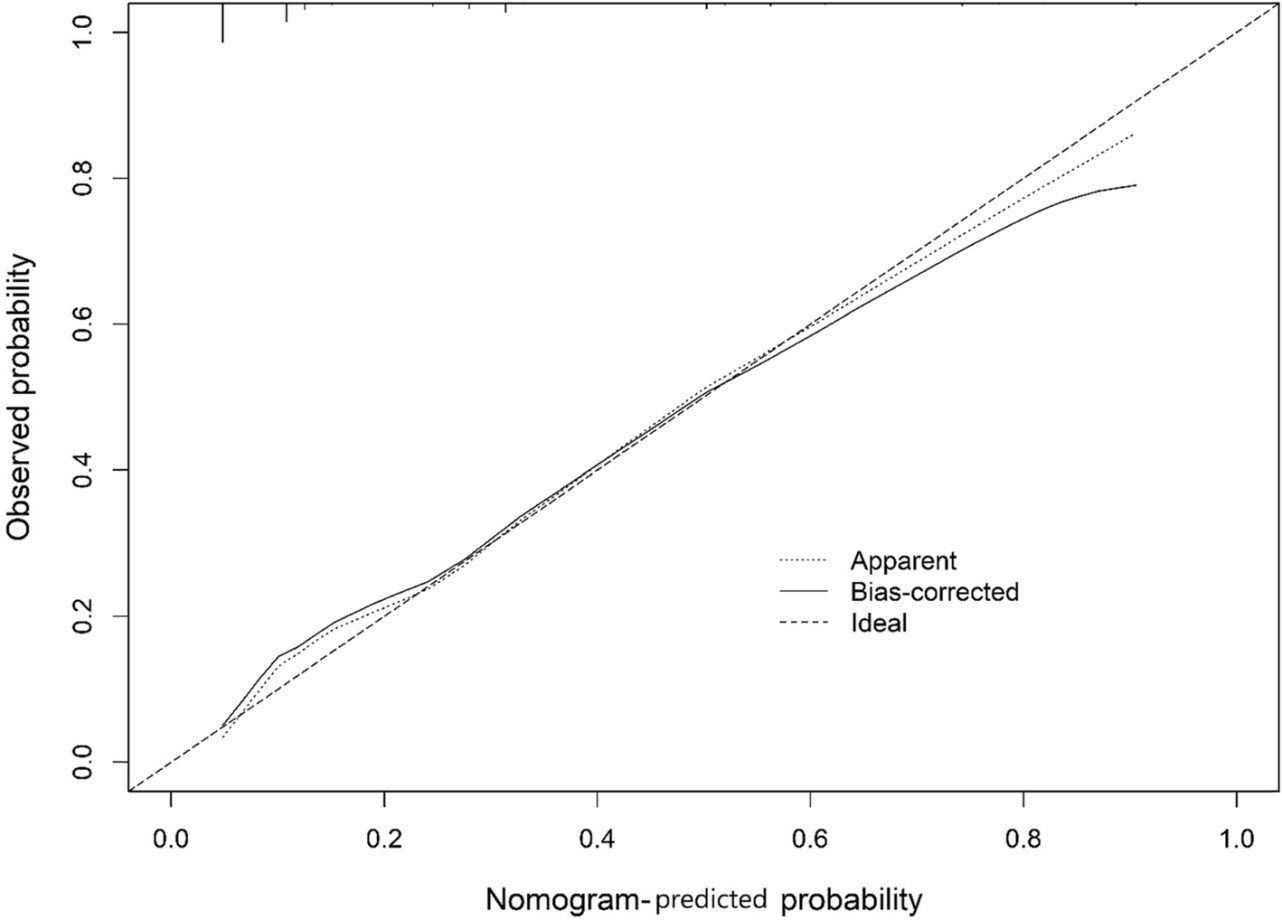
Calibration curve of prediction model for postoperative urosepsis following PCNL in patients with DM. DM = diabetes mellitus. PCNL = percutaneous nephrolithotomy

**Fig. 4:**
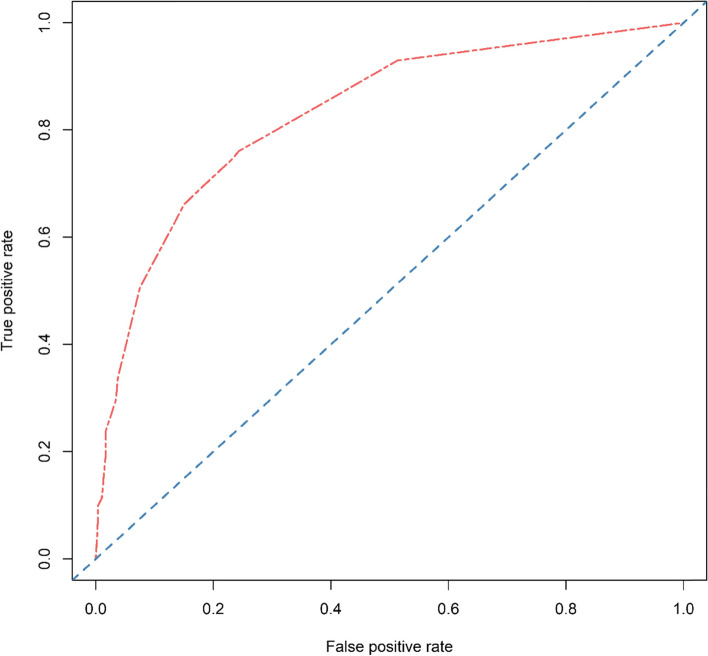
ROC curve of prediction model for postoperative urosepsis following PCNL in patients with DM (AUC = 0.831). AUC = area under the curve. DM = diabetes mellitus. PCNL = percutaneous nephrolithotomy. ROC = receiver operating characteristic

Based on the above results, we used urine culture, urinary nitrite, intraoperative hypotension, and staghorn calculi as variables to construct the nomogram (Fig. 2). The bootstrap method was repeated 1000 times to verify the nomogram model internally. The calculated C-index was 0.824, the calibration curve was drawn, and the calibration curve of the nomogram model was close to the standard curve (Fig. 3), and the Hosmer–Lemeshow goodness-of-fit test was performed (*P* = 0.972 > 0.05). The AUC of the model was 0.831 (Fig. 4), which indicates that the accuracy of the nomogram model predicts the probability of urosepsis after PCNL is good.

## Discussion

The main aim of our study was to find out the risk factors associated with the urosepsis after PCNL in patients with diabetes. On this basis, a new nomogram was constructed and validated to predict urosepsis after PCNL. Currently, this is a completely new logistic regression analysis of patients with diabetes mellitus undergoing PCNL surgery combined with intraoperative blood pressure, and the risk factors can be obtained immediately after surgery to assess the risk of urosepsis early to provide early intervention and treatment.

PCNL is a minimally invasive and effective method for the treatment of kidney stones. It is usually the first choice for treatment of complex upper urinary stones [1]. However, this treatment still has the potential risk of complications, and its incidence rate is as high as 8% [10]. Stone colonization bacteria released during PCNL translocate through systemic circulation, resulting in postoperative infection and even sepsis [11]. Once the urosepsis worsens after surgery, it is not treated promptly and may lead to life-threatening complications, such as septic shock and multiple organ dysfunction. Therefore, through the early identification of the factors associated with urosepsis, it is significant to avoid serious postoperative complications. In our study, the incidence rate of postoperative urosepsis was 19.4%, which was higher than that reported in a previous study [5]. This may be related to all patients with diabetes enrolled in this study. Diabetes seems to have many effects on the risk of infection, including abnormal immune responses and increased susceptibility to common complications, such as neuropathy and vascular insufficiency. Hyperglycemia has been shown to impair neutrophil function and T lymphocyte responses to infection. In addition, the function of polymorphonuclear neutrophils in patients with diabetes has been proven to be improved, which may make them more susceptible to infection [12, 13]. In short, this is mainly due to the decline in immunity caused by diabetes and the proliferation of urinary bacteria. Therefore, for patients with diabetes undergoing PCNL, we need to determine the independent risk factors related to postoperative urosepsis from preoperative and intraoperative factors to identify and intervene early.

In this study, intraoperative hypotension was an independent risk factor for predicting urosepsis after PCNL in patients with diabetes (OR = 8.968, *P* < 0.001). Intraoperative hypotension events increase the probability of urosepsis in postoperative patients by 8.9 times, which highlights the important role of anesthesiologists in intraoperative management. Although there is no relevant study to confirm the association between the duration of hypotension and postoperative urosepsis, the association between duration and other complications has been reported [14]. We hypothesized that hypotension is closely associated with renal perfusion. When intraoperative blood pressure decreases, the effective glomerular filtration function decreases, resulting in a prolonged residence time of bacteria in renal urine, which increases the incidence of postoperative infection complications. It is the responsibility of anesthesiologists to determine and manage intraoperative hypotension over time. Transforming this clue into a visual score can further guide the treatment of urosepsis that may occur postoperatively.

This study also found that the risk of urosepsis in patients with positive urine nitrite before operation was 3.33 times that in patients with negative urine nitrite (*P* = 0.007). Urine nitrite is an important basis for the determination of urinary infection, which is helpful for the early diagnosis and treatment of urinary tract infection. Similarly, in Chen’s study, urine nitrite positivity was also an independent risk factor for urosepsis after PCNL [15].

Urine culture positivity was another risk factor(OR = 2.123, *P* = 0.023). We have known that the clinical significance of preoperative midstream urine bacterial culture is important. Gutierrez studied 865 patients undergoing percutaneous nephroscopy and found that a positive preoperative middle urinary culture (OR = 2.12, 95% confidence interval [1.69–2.65]) was related to the fever after PCNL [16]. Therefore, to avoid urosepsis in patients undergoing PCNL with positive preoperative urine culture, antibiotic sensitivity must be accurately used before surgical treatment.

Rivera analyzed a number of infectious factors after PCNL and found in a multivariate analysis that only staghorn stones were independently associated with an increased risk of fever/SIRS/urosepsis (OR = 3.14, *P* = 0.02) [17]. The same conclusion was reached in patients with diabetes undergoing PCNL. Using logistic regression analysis, Wei et al. showed that diabetic comorbidities and staghorn stones were independent risk factors for postoperative infectious complications [18]. Similarly, in our study, the risk of urosepsis in patients with staghorn calculi was 3.180 times that in patients with non-staghorn calculi (*P* = 0.002).

Based on the relative risk of each factor, namely preoperative urine culture, positive urine nitrite, staghorn calculi, and intraoperative hypotension, we constructed a nomogram able to predict postoperative urosepsis. Through internal verification, the C-index of the nomogram was 0.824, and the AUC of the model was 0.831, indicating good consistency. The above four risk factors can be determined for the first time after the operation. Therefore, when planning an operation, we need to fully consider the probability of urosepsis in patients with high risks. This prediction model can not only help in clinical decision-making but also provide timely and reliable evidence for patients with potentially severe infections and a visual instrument for postoperative infection assessment. It is convenient for doctors and patients to communicate with PCNL and possible risks, which is very important for the perioperative management of patients with diabetes. The study is also an attempt to include an in-depth discussion on the probability of urosepsis in combination with intraoperative hemodynamics. We also recommend that anesthesiologists pay more attention to the patient’s vital signs, remind the physician to end the operation as soon as possible according to the patient’s intraoperative conditions, and provide active antibiotic treatment.

As expected, patients with urosepsis had significantly prolonged hospital stay and overall complication rates. Therefore, to avoid the occurrence of serious complications and shorten hospital stay, we recommend that for patients with diabetes scheduled to undergo PCNL, the possibility of postoperative urosepsis should be evaluated in detail and the intraoperative hemodynamic conditions of patients should be considered. Furthermore, if the possibility of urosepsis is high, active treatment should be performed as soon as possible.

This study has some limitations. This was a retrospective study conducted at a single institution. In addition, the sample size was small, probably because we mainly evaluated patients with diabetes. In addition, our nomogram was based on several readily available predictors. Due to the long time span of this study and the limitations of medical care in China, this study did not include some new indicators, such as procalcitonin and C-reactive protein. Despite these limitations, to the best of our knowledge, the nomogram constructed in this study is of great significance in the early identification of patients at risk of urosepsis.

## Conclusions

In conclusion, positive urine culture, positive urine nitrite, staghorn calculi, and intraoperative hypotension were independent risk factors for urosepsis after one-stage PCNL for upper urinary calculi in patients with diabetes. We constructed a prediction model to predict postoperative urosepsis, and this new nomogram could accurately determine the probability of urosepsis after PCNL in patients with diabetes.

## Data Availability

The datasets used and analysed during the current study are available from the corresponding author on reasonable request.

## Abbreviations

DM: Diabetes mellitus
PCNL: Percutaneous nephrolithotomy
T2DM: Type 2 diabetes mellitus
2-h PG: Two-hour 75-g postload plasma glucose level
OGTT: Oral glucose tolerance test
FPG: Fasting plasma glucose
ADA: American Diabetes Association
CT: Computed tomography
WBC: White blood cells
SIRS: Systemic inflammatory response syndrome
NIT: Urine nitrite
AUC: Area under the curve
BMI: Body mass index
OR: Odds ratio
